# Comparative transcriptome analysis of obligately asexual and cyclically sexual rotifers reveals genes with putative functions in sexual reproduction, dormancy, and asexual egg production

**DOI:** 10.1186/1471-2164-14-412

**Published:** 2013-06-19

**Authors:** Sara J Hanson, Claus-Peter Stelzer, David B Mark Welch, John M Logsdon

**Affiliations:** 1 Department of Biology and Interdisciplinary Program in Genetics, University of Iowa, 301 Biology Building, Iowa City, IA 52242, USA; 2 Institute for Limnology, University of Innsbruck, Herzog Odilostrasse 101, A-5310, Mondsee, Austria; 3 Josephine Bay Paul Center, Marine Biological Laboratory, 7 MBL Street, Woods Hole, MA 02543, USA

**Keywords:** Evolution of sexual reproduction, Differential expression analysis, Gene ontology analysis, Meiosis, Gametogenesis, Resting eggs, Mixis induction

## Abstract

**Background:**

Sexual reproduction is a widely studied biological process because it is critically important to the genetics, evolution, and ecology of eukaryotes. Despite decades of study on this topic, no comprehensive explanation has been accepted that explains the evolutionary forces underlying its prevalence and persistence in nature. Monogonont rotifers offer a useful system for experimental studies relating to the evolution of sexual reproduction due to their rapid reproductive rate and close relationship to the putatively ancient asexual bdelloid rotifers. However, little is known about the molecular underpinnings of sex in any rotifer species.

**Results:**

We generated mRNA-seq libraries for obligate parthenogenetic (OP) and cyclical parthenogenetic (CP) strains of the monogonont rotifer, *Brachionus calyciflorus*, to identify genes specific to both modes of reproduction. Our differential expression analysis identified receptors with putative roles in signaling pathways responsible for the transition from asexual to sexual reproduction. Differential expression of a specific copy of the duplicated cell cycle regulatory gene *CDC20* and specific copies of histone *H2A* suggest that such duplications may underlie the phenotypic plasticity required for reproductive mode switch in monogononts. We further identified differential expression of genes involved in the formation of resting eggs, a process linked exclusively to sex in this species. Finally, we identified transcripts from the bdelloid rotifer *Adineta ricciae* that have significant sequence similarity to genes with higher expression in CP strains of *B. calyciflorus*.

**Conclusions:**

Our analysis of global gene expression differences between facultatively sexual and exclusively asexual populations of *B. calyciflorus* provides insights into the molecular nature of sexual reproduction in rotifers. Furthermore, our results offer insight into the evolution of obligate asexuality in bdelloid rotifers and provide indicators important for the use of monogononts as a model system for investigating the evolution of sexual reproduction.

## Background

Understanding the origins and persistence of sexual reproduction have long been recognized as challenges in evolutionary biology [1-3]. Although sexual reproduction is pervasive in eukaryotes [4]—suggesting it provides critical benefits to an organism—it is also frequently and sporadically lost [e.g. [5,6]]—suggesting it can be dispensable. In fact, loss of sexual reproduction appears to provide several immediate benefits to a lineage, including a two-fold increase in the production of females, the preservation of beneficial combinations of alleles [3], and the loss of risky and energetically expensive processes and behaviors associated with mating. Dubbed “the queen of problems in evolutionary biology” [2], a comprehensive explanation for the prevalence of sexual reproduction in nature has remained elusive [7-9].

Rotifers—a diverse phylum of aquatic invertebrates—are an interesting example of the diversity in reproductive modes found in Metazoa: they include obligate sexual (seisonids and acanthocephalans), facultative sexual (monogononts), and obligate asexual (bdelloids) lineages. Indeed, bdelloid rotifers have persisted and diversified into hundreds of species over tens of millions of years without the canonical forms of sexual reproduction [10,11]. However, it has been difficult to establish clear expectations for any potential cryptic forms of sex in bdelloids without first understanding the molecular nature of sex in relatives such as the facultatively sexual monogononts, from which bdelloids diverged over 40 million years ago [11].

**Figure 1:**
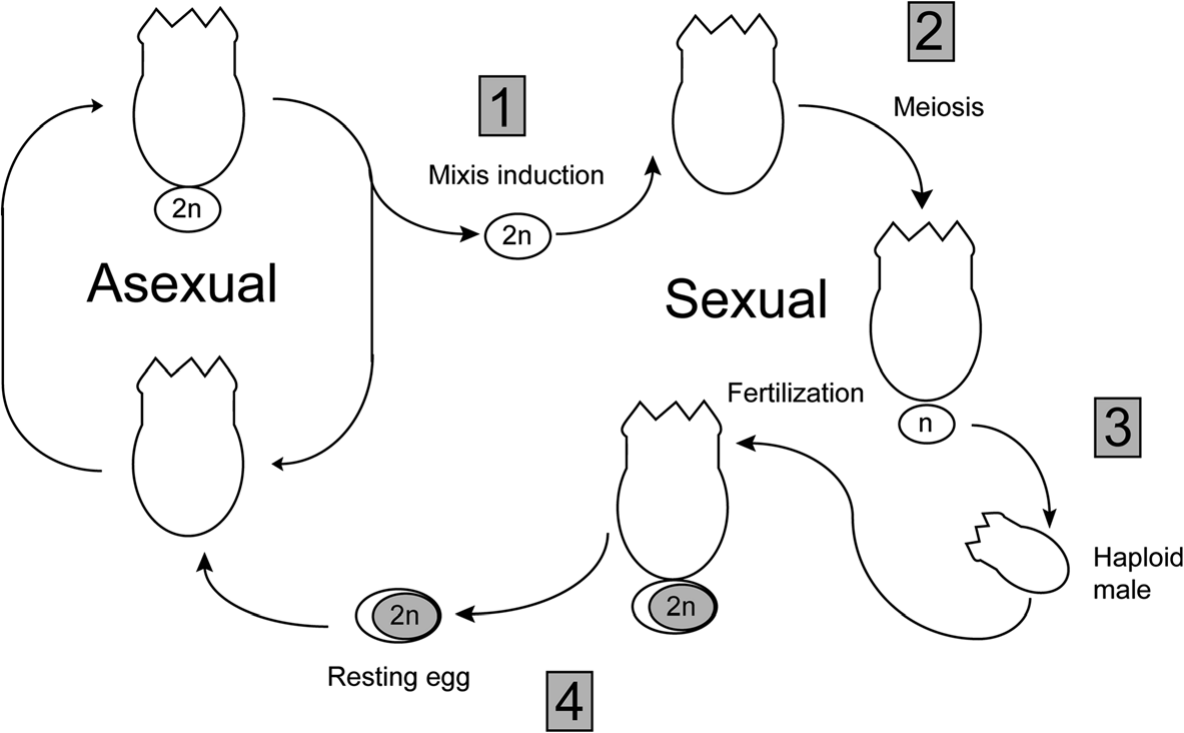
**Diagram of monogonont rotifer life cycle.** Asexual females undergo ameiotic oogenesis to produce diploid eggs. Upon encountering a mixis induction cue (1), asexual females produce sexual daughters who produce eggs *via* meiosis (2). When unfertilized, these haploid eggs develop into males (3) who fertilize sexual females to produce diploid resting eggs (4). Resting eggs develop into asexual females.

Monogonont rotifers have short generation times (~2-3 days) and are useful models for several areas of study, including the evolution of sex, aging, and toxicology [12]. As cyclical parthenogens, monogononts alternate between generations of asexually and sexually reproducing females (Figure 1). The trigger for the switch from asexual (amictic) to sexual (mictic) reproduction for many monogonont species is a small peptide secreted by females [13,14]. Once a threshold concentration of this mixis induction peptide is reached, females are produced that generate haploid eggs by meiosis; these eggs give rise to haploid males when unfertilized and diploid resting eggs when fertilized. The resting eggs, which are initially dormant and can survive cold and dry conditions, develop into amictic females [15].

Intriguingly, the sensitivity of monogononts to the chemical signal and transition to sexual reproduction is highly polymorphic and can be lost entirely. Sex loss has been observed in several monogonont species subjected to long-term laboratory culture [16,17] and is mediated by a single locus in the monogonont *Brachionus calyciflorus*[18]. These *B. calyciflorus* strains reproduce exclusively by obligate parthenogenesis (OP) and no longer respond to the chemical signal that induces mixis in cyclical parthenogenetic (CP) strains.

OP and CP strains of *B. calyciflorus* provide a powerful means through which the evolution of sexual reproduction can be directly tested by allowing direct competition between sexually and asexually reproducing populations [19]. However, little is known about the molecular underpinnings of sexual reproduction in monogononts generally, or the underlying mechanism for the loss of sex in *B. calyciflorus* specifically. Furthermore, several key processes are linked exclusively with sexual reproduction in monogononts and are therefore lost in the OP strains (Figure 1). First, the signaling pathway resulting in the induction of sexual reproduction has apparently been disrupted in the OP strains. Although progesterone signaling contributes to sex induction in the monogonont *Brachionus manjavacas*[20,21], *B. calyciflorus* does not appear to respond to the same cues [22]. Comparisons between OP and CP strains may therefore help elucidate the pathway in *B. calyciflorus*, allowing an examination of the divergence of this signaling pathway in monogononts. Second, production of haploid eggs through a reductive division (meiosis) and the presence of males are unique to sexual populations. Identification of genes specific to meiosis or the production of males through OP and CP expression patterns will offer insights into these processes in monogononts, which will provide an important framework for the study of the asexual bdelloids, as purifying selection should no longer be acting on these loci once sex has been lost. Finally, resting eggs—which are capable of surviving harsh environmental conditions and are used for overwintering—are produced only by fertilization. Previous studies have established gene expression associated specifically with resting eggs in *Brachionus plicatilis*[23,24]; however, genes involved in the early stages of oogenesis and egg development have not been examined quantitatively.

In order to elucidate the gene expression changes associated with sexual reproduction in monogononts, we generated transcriptome data from OP and CP strains of *B. calyciflorus*. We observed increased expression of genes associated with steroid signaling, meiosis, gametogenesis and dormancy in the CP strains, implicating specific pathways in processes related to sex in *B. calyciflorus.* Further, several genes with increased expression in the OP strains were identified, suggesting pathways specific to asexual egg production. Finally, we analyzed previously published bdelloid gene expression data in the context of the observed monogonont expression patterns in order to identify genes present in the bdelloids that have putative roles in monogonont reproduction. Our findings provide molecular insights into sexual reproduction in monogononts and apply an initial comparative approach to understanding the nature of reproduction in bdelloids.

## Methods

### RNA extraction and library preparation

OP and CP clones of *Brachionus calyciflorus* homozygous for the *op* and wild-type alleles, respectively, were generated from the same heterozygous mother by selfing [18]. Six *B. calyciflorus* females were added to 600 mL cultures of 5 × 10^5^ cells/mL *Chlamydomonas reinhardtii* in COMBO medium [25]. The cultures grew at 25 C and with 24 hour light exposure for ~6 days. Density of females was determined every day by removal of ~10 mL of culture and counting individual females under a microscope at low magnification. When cultures reached a density of ~25-40 females/mL, they were harvested for RNA. To confirm the presence of sexually-reproducing females in the CP cultures, a sample of females was used to determine rate of mixis induction as described previously [26], and the absence of males and resting eggs was confirmed for the OP cultures. The rotifer cultures were filtered through 20 μm Millipore nylon filters to remove algal cells, the rotifers were washed in COMBO medium, and rinsed into 15 mL conical tubes. Total RNA was isolated using Trizol reagent (Ambion, Life Technologies, Grand Island, NY) according to manufacturer’s instructions. RNA quality was determined by Experion Automated Electrophoresis (Bio-Rad Laboratories, Hercules, CA) according to manufacturer’s instructions before construction of mRNA-seq libraries using the mRNA-seq Sample Prep kit (Illumina, Inc., San Diego, CA). Seventy-six base pair single end reads were sequenced from each library on an Illumina Genome Analyzer IIx platform at the Iowa State University DNA Facility (http://www.dna.iastate.edu/nextgen.html).

### Illumina sequence analysis

Analysis of Illumina sequence reads was performed in part using the Galaxy server (http://galaxyproject.org/) [27-29]. Adapter sequences were trimmed from the ends of the reads, and a blastn search revealed reads that matched *C. reinhardtii* transcripts [30]. Reads with an alignment length ≥ 20 base pairs matching an algal sequence were removed from the libraries. Transcriptome assembly was performed using the Tuxedo pipeline, where reads were mapped to a partial assembly of the *B. calyciflorus* genome [31]*via* Tophat [32], assembled into transcripts using Cufflinks (assembly for individual samples with 0.1 minimum isoform fraction and 0.1 pre-mRNA fraction) and Cuffmerge (combined assembly from all samples). Differential expression was determined between OP and CP samples with Cuffdiff using a minimum alignment count of 500 [33,34]. Differential expression between OP and CP samples was also determined with edgeR using a table of counts constructed with the fragments *per* kilobase locus length *per* million reads mapped (FPKM) values determined by Cufflinks [34]. Gene ontology mapping and enrichment analyses were performed with BLAST2GO (http://www.blast2go.com/b2ghome). The transcriptome assembly projects have been deposited at GenBank under the accessions GACQ00000000 and GACL00000000. The versions described in this paper are the first versions, GACQ01000000 and GACL01000000. Assembled transcripts shorter than 200 base pairs are given in Additional files 1, 2, 3 and 4.

### Library validation

OP and CP RNA samples used for Illumina library construction were treated with DNase I prior to first-strand cDNA synthesis using Superscript II reverse transcriptase (Invitrogen, Life Technologies, Grand Island, NY). Quantitative reverse transcriptase polymerase chain reaction (qRT-PCR) was performed with Platinum SYBR Green quantitative PCR (qPCR) Supermix (Invitrogen, Life Technologies, Grand Island, NY) and a 1:25 dilution of the cDNA. Primers designed for amplification of thirteen genes from cDNA in the qPCR reaction (200 nM final concentration) are listed in Additional file 5. Relative quantification of gene expression was determined for each gene, with actin transcript amplification used for normalization.

### Phylogenetic analysis

For analysis of histone H2A, amino acid sequences of metazoan and fungal homologs were obtained from the National Center for Biotechnology Information (NCBI) database by keyword search and aligned with *B. calyciflorus* sequences by ClustalX version 2.1 [35]. Alignments were manually curated in MacClade (http://macclade.org/index.html) and phylogenetic analysis was performed using the Bioportal parallel computational resource [36]. Maximum likelihood analysis was performed using PhyML [37] with Whelan and Goldman (WAG) substitution model, best tree topology search, and one thousand bootstrap replicates.

### Bdelloid sequence analysis

Transcript sequences for *Adineta ricciae* were obtained from GenBank (Accession numbers: HE687510 to HE716431). *A. ricciae* transcripts were used as queries against the assembled *B. calyciflorus* transcriptome using tblastx [38] with a Bit Score cutoff of 50. Gene ontology term enrichment was performed using BLAST2GO [39].

## Results and discussion

**Figure 2:**
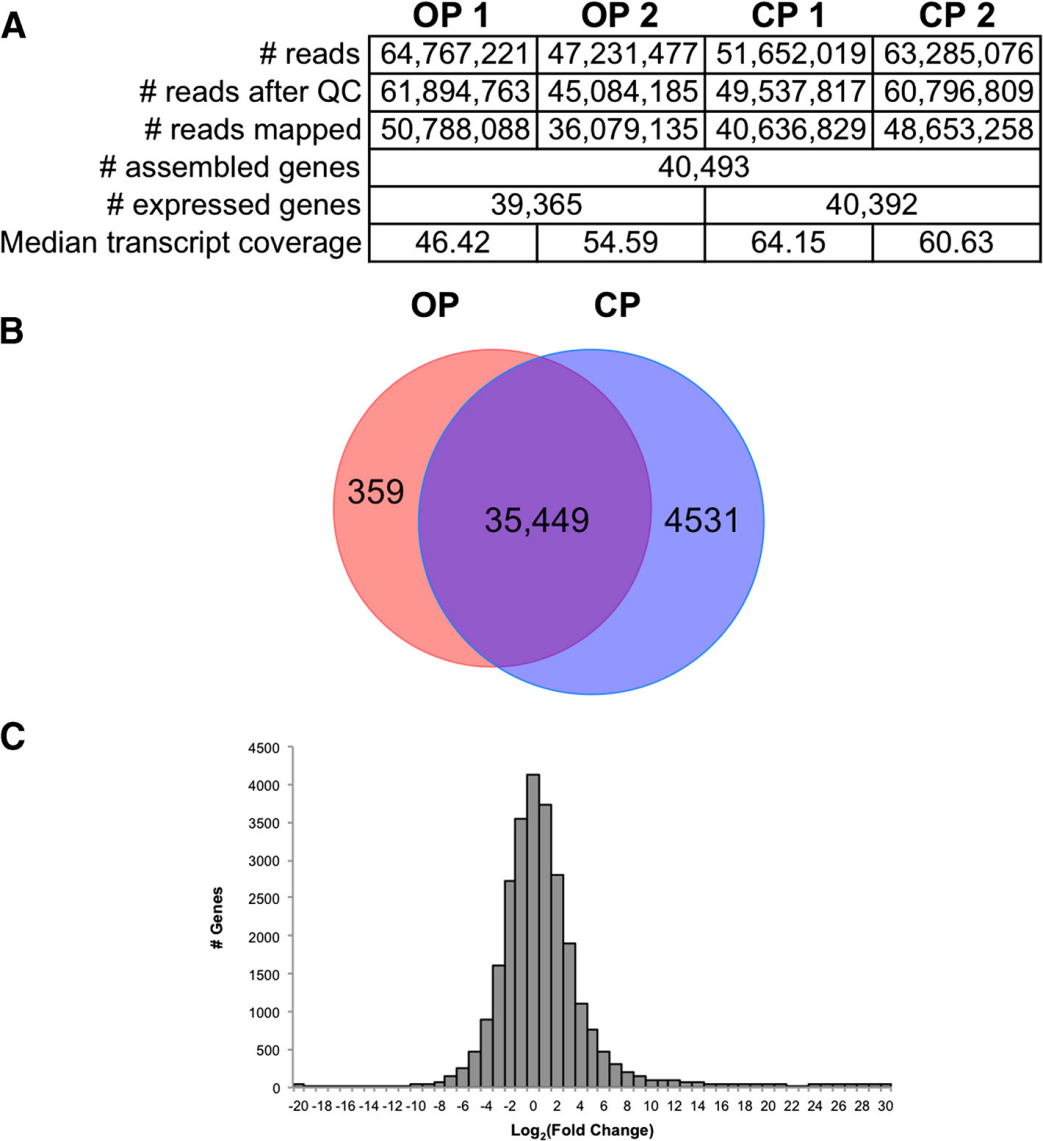
**Comparison of gene expression in OP and CP libraries. A**) Mapping and assembly statistics for OP and CP replicates. QC = quality control (see Methods); FPKM = fragments (*i.e.* reads) per kilobase exon per million mapped reads. For genes assembled using Tuxedo pipeline, **B**) Venn diagram showing genes with an FPKM value of 1 in OP and CP libraries. **C**) Log_2_ (Fold Change) distribution (CP/OP) is shown as determined by edgeR.

To examine global gene expression patterns associated with sexual reproduction in monogononts, we generated mRNA-seq libraries using RNA isolated from two independent cultures of OP and CP populations of *B. calyciflorus*. More than 95% of the generated reads were retained after our quality control measures (adapter clipping and contaminant filtering, see Methods), and these reads were assembled into transcripts and genes using the Tuxedo pipeline (Figure 2A). Although the two OP replicates were similar, the two CP replicates varied considerably in their FPKM values (Additional file 6), which may be due to the complexity of the sampled CP populations (containing asexual females, sexual females, males, amictic eggs, and resting eggs) relative to the OP populations (containing only asexual females and amictic eggs). However, a multi-dimensional scaling analysis in which the distance between samples was determined by the estimated dispersion (performed in edgeR) found for the number of reads mapped to each Cuffmerge-assembled gene clearly separated the two OP samples from the two CP samples (Additional file 6). We therefore continued downstream analyses using the CP samples as biological replicates.

To compare overall gene expression between the OP and CP samples, we first compared the genes expressed in each. Genes with FPKM values greater than 1.0 were determined for both the OP and the CP libraries (Figure 2B). There was a large amount of overlap between the OP and CP samples for these two sets of genes (~88%), demonstrating that the vast majority of genes with at least a moderate level of expression are expressed in both strains of *B. calyciflorus*. Furthermore, several of the most highly expressed genes in each strain were also shared (Table 1). Among the genes with highest expression in both OP and CP populations were sequences matching the four mate recognition pheromone genes (MRP) characterized in the monogonont *B. manjavacas*. MRP is a glycoprotein on the surface of females that is detected by male monogononts, establishing a copulation preference for young, conspecific females [40]. Because MRP is expressed in both OP and CP populations, the function of this pheromone in mate recognition may be secondary to a separate, constitutive function, as sexual females are absent in the OP populations. The high level of expression of MRP in both CP and OP populations is consistent with the lack of copulation preference demonstrated by males for sexual females over asexual females in a population [41].

**Table 1:** Assembled genes with highest expression values in OP and CP libraries

**Gene**	**Sequence (top hit)**	**Species**	**Accession**	**E value**
*OP samples*				
contig30046_1_155	MRP motif repeat 3 protein precursor	*Brachionus manjavacas*	ACV04429.1	5.E-12
contig79431_1_261	MRP motif repeat 4 protein precursor	*Brachionus manjavacas*	ACV04427.1	8.E-20
contig101675_1_126	alpha-tubulin	*Chironomus duplex*	AAR97873.1	3.E-19
contig93682_1_197	alpha-tubulin	*Rattus norvegicus*	EDM01432.1	3.E-42
contig62222_1_158	60S ribosomal protein L4	*Pediculus humanus corporis*	EEB17109.1	5.E-16
contig77623_1_272	MRP motif repeat 3 protein precursor	*Brachionus manjavacas*	ACV04429.1	2.E-26
contig28535_1_216	MRP motif repeat 3 protein precursor	*Brachionus manjavacas*	ACV04429.1	6.E-30
contig79401_24_340	MRP repeat motif 5 protein precursor	*Brachionus manjavacas*	ADU18105.1	7.E-28
contig46700_2_86	tropomyosin isoform 5	*Bombyx mori*	ABF51445.1	3.E-03
contig59544_1_116	ribosomal protein L4	*Alligator mississippiensis*	AEK51829.1	2.E-20
contig66153_2771_3902	60S ribosomal protein L23a	*Brachionus plicatilis*	CAQ37753.1	5.E-19
contig06643_1320_1619	ubiquitin/ribosomal S27A fusion protein 3	*Aplysia californica*	AAM09680.1	8.E-27
contig65842_1_441	alpha-tubulin	*Drosophila persimilis*	EDW38107.1	2.E-65
contig59965_1450_2062	60S ribosomal protein L38	*Brachionus plicatilis*	CAQ37756.1	2.E-22
contig60736_1_280	ribosomal protein rps23	*Arenicola marina*	ABW23151.1	4.E-13
contig52695_1_821	60S ribosomal protein L35	*Epinephelus coioides*	ADG29172.1	8.E-34
contig11838_1_791	60S ribosomal protein L15	*Acromyrmex echinatior*	EGI70568.1	3.E-43
contig53952_1_403	60S ribosomal L7a-like protein	*Phragmatopoma lapidosa*	ADW95788.1	1.E-36
contig59138_1_298	60S acidic ribosomal protein P1	*Stomoxys calcitrans*	ACN69158.1	2.E-15
contig04863_1_209	ribosomal protein S18	*Drosophila willistoni*	EDW74864.1	7.E-05
*CP samples*				
contig30046_1_155	MRP motif repeat 3 protein precursor	*Brachionus manjavacas*	ACV04429.1	5.E-12
contig79431_1_261	MRP motif repeat 4 protein precursor	*Brachionus manjavacas*	ACV04427.1	8.E-20
contig101675_1_126	alpha tubulin	*Chironomus duplex*	AAR97873.1	3.E-19
contig93682_1_197	alpha tubulin	*Rattus norvegicus*	EDM01432.1	3.E-42
contig81910_1_195	MRP motif repeat 3 protein precursor	*Brachionus manjavacas*	ACV04429.1	3.E + 00
contig28535_1_216	MRP motif repeat 3 protein precursor	*Brachionus manjavacas*	ACV04429.1	6.E-30
contig79401_24_340	MRP repeat motif 5 protein precursor	*Brachionus manjavacas*	ADU18105.1	7.E-28
contig77623_1_272	MRP motif repeat 3 protein precursor	*Brachionus manjavacas*	ACV04429.1	2.E-26
contig52695_1_821	60S ribosomal protein L35	*Epinephelus coioides*	ADG29172.1	8.E-34
GHICQ5101EO836_17_154	polyubiquitin precursor	*Suberites domuncula*	CAA72800.1	2.E-21
contig90319_1_114	Gram-negative binding protein 7, partial	*Daphnia arenata*	AFI40165.1	3.E-05
contig59965_1450_2062	60S ribosomal protein L38	*Brachionus plicatilis*	CAQ37756.1	2.E-22
contig78014_9_305	hypothetical protein	*Branchiostoma floridae*	EEN52859.1	3.E-03
contig46700_2_86	tropomyosin isoform 5	*Bombyx mori*	ABF51445.1	3.E-03
contig06643_1320_1619	ubiquitin/ribosomal S27A fusion protein	*Aplysia californica*	AAM09680.1	8.E-27
contig04863_1_209	ribosomal protein S18	*Drosophila willistoni*	EDW74864.1	7.E-05
contig82637_1_255	60S ribosomal protein L12	*Brachionus plicatilis*	CAQ37748.1	9.E-46
contig60736_1_280	ribosomal protein rps23	*Arenicola marina*	ABW23151.1	4.E-13
contig65842_1_441	alpha tubulin	*Drosophila persimilis*	EDW38107.1	3.E-65
contig63886_822_2083	voltage-dependent calcium channel beta subunit	*Strongylocentrotus purpuratus*	XP_790453	1.E-05

Genes that had a significant BLAST hit in NCBI are shown.

Notably, we found that a large number of the genes assembled by Cuffmerge (45%) did not have identifiable homologs by BLAST search (e value < 1). In many cases this is likely due to the short length of the assembled sequences, as only 24% of the genes >400 base pairs in length did not have significant BLAST hits. This proportion of unique sequences is consistent with that observed for expression data from the monogonont *B. plicatilis*[42,43]. Furthermore, a recently published transcriptome for the bdelloid rotifer *Adineta ricciae* found that 53% of transcripts did not have a significant BLAST hit [44], which may indicate that the lack of identifiable homologs may be due to limited taxonomic sampling in the NCBI database.

We further examined differential expression between the OP and CP samples using the Bioconductor package edgeR and the Tuxedo software Cuffdiff. Fold change calculations obtained with both Cuffdiff and edgeR for a set of fourteen genes were validated by qRT-PCR (Additional file 7). The distribution of the fold change calculations (Figure 2C, Additional file 8) suggests more genes have higher expression values in CP samples compared with OP samples. Consistent with this observation, more genes were found to have significantly higher expression in Cuffdiff or edgeR (FDR adjusted P-value ≤ 0.05) in the CP than the OP samples (Additional file 9). To determine whether this fold change distribution was due to a generalized decrease in gene expression in the OP strains [e.g. due to dwarfing [18]], we compared the FPKM distribution between the OP and CP libraries (Table 2, Additional file 10). Although the distributions of FPKM values were significantly different between OP and CP when all genes were taken into account, there was no significant difference between OP and CP when a subset of genes with housekeeping functions were compared (Table 2, Additional files 10 and 11). This suggests that increased distribution of FPKM values in the CP libraries is due to increased expression of specific loci rather than an overall increase in the expression of all genes.

**Table 2:** Comparison of FPKM distributions between OP and CP libraries

**Genes**	**Sample**	**OP**	**OP**	**CP**	**CP**	**Kolmogorov-Smirnov**	**Mann–Whitney**
	**size**	**mean**	**variance**	**mean**	**variance**	**value**	**value**
All	39265	1.0320	0.7431	1.4521	0.5545	0.0001	-
Housekeeping	176	2.5819	1.7160	2.5751	1.3709	0.0540	0.4660
Dormancy	200	1.5381	0.9866	2.0287	0.6013	0.0001	-
Meiosis Inventory	107	1.3204	0.4265	1.6428	0.3424	0.0002	-
MI-Recombination	26	0.9094	0.2261	1.3591	0.2432	0.0019	-
MI-Chromosome Struc.& Int.	33	1.4567	0.2878	1.7927	0.1487	0.0328	-
MI-Cell Cycle	48	1.4503	0.5268	1.6950	0.4855	0.0074	-
Nuclear Receptors	73	0.8888	0.7537	1.2949	0.3400	0.0099	-
Male Gametogenesis	39	1.2196	0.4508	1.5884	0.2337	0.0078	-

Mean and variance are given for log-transformed FPKM values for sets of genes examined in this study. Distributions of expression were compared using the Kolmogorov-Smirnov test with statistically similar distributions compared *post-hoc* by Mann–Whitney test.

We next examined the expression of genes involved in several aspects of monogonont reproductive biology in our expression libraries for the OP and CP samples. These include genes with putative roles in mixis induction, meiosis and gametogenesis, dormancy, and asexual egg production.

### Mixis induction

The trigger for the transition from asexual to sexual reproduction in many species of monogononts is a secreted protein signal that accumulates in their environment [14]. Although little is known about the signal or the pathway(s) it activates to induce the transition, several lines of evidence indicate that it is mediated by steroid hormone signaling. First, isolation of the protein signal and sequencing of its N-terminus revealed that its sequence in the species *B. plicatilis* is similar to the mammalian steroidogenesis-inducing protein [14]. Second, exposure to hormones, including estradiol-17 and progesterone, resulted in increased production of sexual females in populations of *B. plicatilis*[21,45]. Third, RNAi knockdown of the progesterone receptor in *B. manjavacas* reduced production of sexual females [20]. Intriguingly, there appears to be species-specificity in the response to steroid hormones, as sexual reproduction is not induced by progesterone treatment alone in *B. calyciflorus* cultures [21,22].

Steroid hormone signaling is mediated by members of the nuclear receptor superfamily, a large family of proteins that are well-conserved across Metazoa [46]. Nuclear receptors have critical functions in developmental processes, including the induction of meiosis during oogenesis in mammals [47,48]. Members of the nuclear receptor superfamily are transcription factors whose activity is modulated through direct interactions with steroid ligands. Nuclear receptors frequently act as homodimers or heterodimers when modulating transcription of target sequences *via* histone acetylation or deacetylation, including auto-regulating and cross-regulating the transcription of other nuclear receptors [48,49].

We identified 73 genes in our transcriptome analysis with significant sequence identity to the DNA-binding domains of the nuclear receptor superfamily, of which five have significantly higher expression levels in CP populations (Additional file 12). Although we were unable to obtain phylogenetic resolution to confirm the orthology of the receptors (data not shown), these data are consistent with the role of steroid signaling in mixis induction.

We also identified differentially expressed genes with functions and GO term designations associated with steroid metabolic and signaling pathways (Additional file 9). CBP/p300 is a transcription factor with histone acetyltransferase activity that acts as a co-regulator of transcription with nuclear receptor superfamily members [50]. Protein Kinase A modulates nuclear receptor activity *via* phosphorylation and has roles during gametogenesis [51]. Bcl-X mediates apoptosis and is regulated through steroid hormone signaling pathways [52]. These genes provide additional candidates for roles in the transition from asexual to sexual reproduction in *B. calyciflorus*.

### Meiosis, recombination, and gametogenesis

Genes with known roles in meiosis based on evidence in well-studied model systems (e.g. yeast, mouse, and nematodes) were previously annotated in monogononts [31]. The expression levels of these genes are shown in Additional file 11. The distribution of expression values for these genes as a group is significantly different in CP than in OP strains, with a greater median expression value in the CP strain (Table 2, Additional file 10). This difference in expression value distribution is also found in more specific categories of gene function within the meiotic gene inventory (Table 2, Additional files 10 and 11).

Individually, very few of the meiotic genes had significantly different expression between OP and CP libraries. In fact, genes that are “meiosis-specific”, *i.e.* have no known roles outside of meiosis [53], were not differentially expressed or had levels of expression that were too low to make accurate statistical comparisons. However, we identified significant differential expression for one copy of CDC20 (Additional file 11) and three copies of Histone H2A (Table 3), both of which appear to be present in multiple copies in the *B. calyciflorus* genome.

**Table 3:** Histone H2A genes identified in OP and CP libraries

	**Locus**	**BLAST**			**OP FPKM**	**CP FPKM**	**Cuffdiff**		**edgeR**		**# A. ricciae transcripts**	**e value**	**A. ricciae accession**
		**e value**	**Organism**	**RefSeq**			**p value**	**q value**	**p value**	**FDR**			
H2A1	contig00755_2006_2354	1.E-33	*Nasonia vitripennis*	XP_001600014.1	3.03	17.71	0.014	0.124	0.187	1.000	1	3.E-11	HE711327
H2A2	contig03654_1516_2032	1.E-55	*Daphnia pulex*	EFX87610.1	2.27	57.22	0.000	0.000	0.018	0.500	1	7.E-33	HE689758
H2A3	contig07701_0_378	1.E-19	*Adineta vaga*	ACH68793.1	1.76	11.43	No Test		0.094	1.000	-	-	-
H2A4	contig12265_530_1237	1.E-64	*Xenopus laevis*	NP_001086775.1	2803.24	2835.08	0.988	0.994	0.988	1.000	6	2.E-28	HE702063
												2.E-60	HE702590
												2.E-50	HE703234
												4.E-31	HE703984
												7.E-33	HE711821
												4.E-53	HE714976
H2A5	contig23888_34_960	8.E-68	*Xenopus (Silurana) tropicalis*	XP_002935049.1	818.68	1038.78	0.746	0.877	0.847	1.000	4	4.E-38	HE689797
												9.E-20	HE690440
												3.E-21	HE700115
												1.E-27	HE706180
H2A6	contig34004_2741_3299	8.E-66	*Xenopus laevis*	NP_001086775.1	2.54	36.65	0.000	0.005	0.042	0.944	5	7.E-33	HE689758
												2.E-28	HE702063
												8.E-32	HE707507
												8.E-18	HE711090
												3.E-11	HE711327
H2A7	contig39260_829_1351	2.E-64	*Acropora formosa*	P35061.2	243.15	204.85	0.790	0.899	0.740	1.000	-	-	-
H2A8	contig87961_4_298	3.E-51	*Gallus gallus*	XP_003642635.1	8.38	30.09	0.064	0.285	0.360	1.000	2	5.E-46	HE693848
												1.E-16	HE714998
H2A9	contig94637_39_541	3.E-65	*Xenopus laevis*	NP_001086775.1	4.29	47.78	0.001	0.012	0.070	1.000	1	2.E-56	HE711680
H2A10	contig09212_1061-1926	8.E-26	*Mustela putorius furo*	AER99544.1	18.87	12.97	0.569	0.778	0.421	1.000	-	-	-
H2A11	contig19788_3764-5623	7.E-38	*Oreochromis niloticus*	XP_003451495.1	21.08	25.43	0.810	0.909	0.624	1.000	-	-	-
H2AZ1	contig14214_0-584	8.E-56	*Cavia porcellus*	XP_003462309.1	16.17	27.45	0.398	0.671	0.740	1.000	-	-	-
H2AZ2	contig21808_7858_8553	1.E-54	*Ascaris suum*	ADY40521.1	238.30	216.52	0.886	0.945	0.932	1.000	-	-	-

No Test = expression values did not meet threshold required for significance testing.

#### CDC20

Progression through mitosis and meiosis is mediated by the anaphase-promoting complex/cyclosome (APC/C), which acts as a ubiquitin ligase targeting proteins for proteasomal degradation. APC/C activity is mediated by CDC20, which has paralogs (Fizzy and Fizzy-Related) functioning in both mitosis and meiosis [54]. In addition, an arthropod-specific paralog, Cortex, functions exclusively in meiosis [55].

We previously identified four copies of CDC20 in the genome of *B. calyciflorus* with phylogenetic analysis placing three copies in the Fizzy clade (CDC20A, B, and C) and one copy in the Fizzy-related clade (CDC20D) [31]. One Fizzy copy (CDC20A) is differentially expressed, with ~18-fold higher expression in the CP populations (Additional file 11). Its higher expression in CP populations suggests it may function in a process specific to sexual reproduction in *B. calyciflorus*, such as resting egg production, spermatogenesis, or meiosis, or is involved in a developmental process specific to the sexual cycle.

#### Histone H2A variants

Histone octamers comprised of the histones H2A, H2B, H3, and H4 are the primary protein component of chromatin. Histones are globular proteins that have a highly conserved core sequence and a C-terminal tail sequence that is post-translationally modified to alter chromatin structure and transcriptional activity. Canonical histones may be replaced by variants in a spatial or temporal manner in response to internal or external cues [56]. In particular, variants of histone H2A are associated with DNA repair and gametogenesis. Histone H2A.X localizes to DNA double-strand breaks, where it recruits components of repair machinery. This occurs when breaks are induced through exposure to endogenous or exogenous agents, as well as in meiosis, when double-strand breaks are created by the topoisomerase Spo11 (Fernandez, 2004). Another histone variant specific to mammals, H2A.bbd, localizes to transcriptionally active sites within mouse testes, where genes with meiotic expression patterns are located (Soboleva, 2011).

Bdelloid rotifers, which apparently reproduce solely through ameiotic parthenogenesis, possess three histone H2A variants, some with multiple identified alleles. These H2A sequences have long C-terminal tail sequences that do not correspond to the tails of canonical or known variants of H2A, including the well-conserved SQE/D sequence in the tail of H2A.X [57]. Although functionally uncharacterized, because of the importance of H2A variants to double-strand break repair in other systems, it is hypothesized that these novel variants may have evolved to aid the bdelloids in DNA repair following bouts of desiccation, which the rotifers can survive at any life stage. This same report identified three apparently canonical histone H2A sequences in the monogonont rotifer *B. plicatilis*[57].

**Figure 3:**
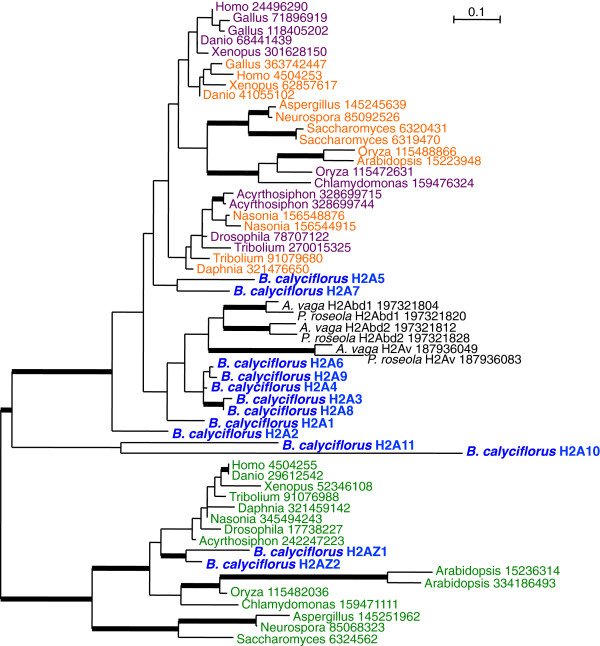
**Histone H2A phylogeny.***B. calyciflorus* H2A sequences are shown in blue, bdelloid H2A sequences are shown in black. Phylogeny of canonical Histone H2A (purple), H2A.X (orange), and H2A.Z (green) based on 142 amino acid sequence alignment. Thickened branches indicate bootstrap support ≥ 70%. NCBI accession numbers for the sequences used in the alignment are given in the tree.

We searched the transcriptome and partial genome assembly for *B. calyciflorus* to identify all histone sequences (Table 3, Additional file 11), and identified 13 histone H2A variants (Figure 3). Previous histone H2A sequence analysis was unable to resolve histone H2A relationships at deeper phylogenetic nodes, although the variant H2A.Z is monophyletic [57]; we found similar results through our analysis. Although H2A.Z was not identified in the previous analysis of monogonont H2A sequences [57], two of the *B. calyciflorus* sequences fall within this clade with strong bootstrap support, suggesting that loss of H2A.Z in rotifers may be limited to the bdelloid lineage. Six *B. calyciflorus* H2A sequences cluster with bdelloids, although there is only moderate bootstrap support (<70%) for this relationship. As is typically observed in H2A genes, no introns were found in 12 of the genes [58], however H2A11 contains three introns, and encodes a much longer C-terminal tail than the other H2A genes in *B. calyciflorus*. A long C-terminal tail has previously been described for macroH2A, a vertebrate-specific H2A variant [59].

Our transcriptome analysis indicated that three of the histone H2A sequences are differentially expressed between OP and CP populations, with higher expression in the CP strains (Table 3). Differential expression of these sequences suggests a role for these H2A genes in processes related to sexual reproduction in monogononts, perhaps similar to the functions performed by H2A.X or H2A.bbd in other systems.

In addition to these novel H2A sequences, we identified two histone interacting genes with significantly higher expression in the CP populations (Additional file 9). Tonsoku interacts with both histone H2A and H2B, and has a role in resolution of stalled replication forks and double strand breaks [60]. Pax-Interacting Protein 1 recruits the H3K4 methyltransferase complex to trimethylate specific sites and activate transcription [61], and is required for both embryonic and somatic tissue differentiation [62]. H3K4 trimethylation is also required during meiosis for pairing of homologs and progression through pachytene [63].

#### Gametogenesis

We identified 39 genes in our study with significant sequence identity to genes with roles in spermatogenesis or sperm morphology (Additional file 13). Several structural components of sperm flagellar tails were identified in the assembled genes from *B. calyciflorus*: Outer Dense Fiber of Sperm Tails 2 [64,65], Sperm-Associated Antigen 6 [66,67], Sperm-Associated Antigen 16 [68], Sperm-Associated Antigen 17 [67], Sperm Flagellar Protein 1 [69] and Sperm Flagellar Protein 2 [70]. We also identified genes with regulatory roles for transcription or cell cycle progression in the testes: AMY-1-associated protein expressed in testis-1 (AAT-1) [71], Nuclear autoantigenic sperm protein [72], Round Spermatid Basic Protein 1 [73], Sperm-Associated Antigen 1 [74,75], Sperm-Associated Antigen 8 [76], Testis Expressed gene 14 [77,78], Testis expressed gene 15, and Testis specific ser/thr protein kinase 2 [79]. Finally, we found several genes whose gene expression and/or localization patterns are specific to testes or sperm in various developmental stages in Metazoa, including Granulin [80], PolyA Polymerase Beta [81], and Spermatogenesis Associated Genes [82-88].

Intriguingly, these genes were expressed in both OP and CP strains and while the distribution of expression of the genes as a group was significantly different between OP and CP with a higher median expression value in CP samples (Table 2, Additional file 10), none of the genes were significantly differentially expressed individually (Additional file 13). The expression of these genes in the presence and absence of males is consistent with a previous study in *B. plicatilis* showing that genes with putatively male-specific functions were also expressed in females [43]. Indeed, several of these genes have been implicated in processes outside of spermatogenesis, such as roles in cilia development and in more generalized differentiation processes including oncogenesis (e.g. [70,78]).

Finally, several genes with other roles in gametogenesis have significantly higher expression in the CP populations (Additional file 9). Brain Tumor Protein is a homolog of Mei-P26, which maintains germline stem cells and promotes germline differentiation during oogenesis in *Drosophila melanogaster* through interactions with the miRNA pathway protein Argonaute 1 [89,90]. HSP70, in addition to an implicated role in monogonont dormancy [23], also plays a role in chromatin restructuring during spermatogenesis [91,92]. Furthermore, Sacsin integrates the HSP70 pathway with proteasomal degradation during gametogenesis [93] and also has higher expression in CP strains.

### Dormancy

Using BLAST2GO, we performed gene ontology (GO) analysis on the genes with significantly different expression levels between OP and CP populations. First, GO terms were assigned to the differentially expressed sequences that had identifiable homologs in NCBI (Additional file 9). GO term enrichment analysis was performed using the annotated Cuffmerge-assembled transcriptome as a reference. The most significantly enriched GO terms are shown in Table 4. Within this set of enriched terms, several are attributable to the presence of resting eggs exclusively in the CP populations. Genes with antioxidant functions have previously been identified in resting egg transcriptomes, including superoxide dismutases and peroxiredoxin [24]. These genes contribute to the enrichment of antioxidant activity (GO:0016209) in the differentially expressed set of genes (Table 4, Additional file 11).

**Table 4:** **Gene ontology enrichment analysis for *B. calyciflorus *genes with higher expression in CP strains**

**GO-ID**	**Term**	**Category**	**FDR**	**P-Value**
GO:0042302	structural constituent of cuticle	F	6.03E-10	1.36E-13
GO:0005576	extracellular region	C	2.89E-08	2.60E-11
GO:0008061	chitin binding	F	1.98E-07	1.93E-10
GO:0030246	carbohydrate binding	F	4.47E-07	5.02E-10
GO:0030247	polysaccharide binding	F	4.79E-07	6.10E-10
GO:0001871	pattern binding	F	4.79E-07	6.10E-10
GO:0006030	chitin metabolic process	P	5.02E-07	6.77E-10
GO:0006022	aminoglycan metabolic process	P	7.09E-07	1.06E-09
GO:0043169	cation binding	F	3.28E-04	6.39E-07
GO:0043167	ion binding	F	4.06E-04	8.21E-07
GO:0008430	selenium binding	F	1.30E-03	2.72E-06
GO:0005976	polysaccharide metabolic process	P	1.73E-03	3.89E-06
GO:0016209	antioxidant activity	F	2.41E-03	6.14E-06
GO:0004252	serine-type endopeptidase activity	F	3.01E-03	8.35E-06
GO:0046914	transition metal ion binding	F	3.17E-03	9.52E-06

*F* molecular function, *C* cellular component, *P* biological process.

Further, enrichment for other terms likely relating to resting egg components/structure was identified, such as chitin binding (GO:0008061) and chitin metabolic process (GO:0006030). Asexually produced eggs are encased in one chitin-containing shell, while resting eggs have two shells, with the inner shell of the resting eggs—also composed of chitin—thicker than the asexual egg shell [43], providing a likely explanation for the enrichment of these terms in the differentially expressed genes. Interestingly, these GO terms were not identified as enriched in the previous resting egg transcriptome analyses [24], which focused on transcripts exclusively in the eggs. This discrepancy may indicate the maternal production of these transcripts during early stages of egg development, prior to egg extrusion.

In addition to significantly different distribution of expression values for dormancy genes as a group in CP samples (Table 2, Additional file 10), we identified several individual genes with significantly higher expression in the CP strains that were previously associated with dormancy in the monogonont *B. plicatilis* (Additional file 11). Aquaporin 3 is involved in water and small molecule transport [94]. Genes involved in lipid metabolism, including cathepsin L, fatty acid binding genes, and lipases may be involved in yolk processing that differs between resting and amictic eggs in *B. calyciflorus*[95,96]. Heat shock proteins are molecular chaperones that prevent the aggregation of misfolded proteins and have been implicated in dormancy in rotifers and other systems [97]. Trehalose is a likely energy source for dormant organisms, suggesting a role for trehalose metabolic enzymes in monogononts [98,99]. Late embryogenesis-abundant genes have been associated with embryonic development and dormancy in several organisms, including rotifers [100,101].

### Asexual reproduction

The number of genes with significantly higher expression in the OP strain was much lower than that for the CP strains (Additional file 9). This is expected given that asexually reproducing females cannot be morphologically distinguished from sexually reproducing females in the CP strains. These females were therefore also included in CP samples, which likely decreased the fold change in expression of any gene specific to asexual females and/or their eggs. However, several interesting genes were found to have significantly higher expression in the OP strains.

#### Transposable elements

Several genes with significant sequence similarity to genes associated with transposable elements show significantly higher expression in OP strains. Transposable element replication is highly regulated through multiple pathways in mammals and *Drosophila*, including transcriptional repression through epigenetic modifications, RNA editing, transcript degradation by RNA interference, and DNA repair pathways [102]. Further, specific developmental stages during gametogenesis and embryonic development in mammals allow windows of opportunity for transposable elements to replicate and integrate into the host genome [102,103]. Increased expression of transposons in OP strains suggests developmental differences that result in reduced suppression of these elements in *B. calyciflorus*.

#### Cytoskeleton

Septins are cytoskeletal components that are flexibly combinatorial in the formation of filaments and rings. In ring form, septins act as scaffolds that aid in cell signaling, diffusion barriers between cells during cytokinesis, and components of cilia and flagella [104]. The increased expression of a gene with significant sequence similarity to Septin 10 in OP strains could indicate a role for cytoskeletal restructuring during asexual female development or oogenesis. This is supported by previous transcriptome data from asexual eggs in *B. plicatilis*, in which several other cytoskeletal components had increased expression [24].

#### Metabolism

Two genes with sequence similarity to pancreatic triacylglycerol lipases were expressed at significantly higher levels in OP strains, while one pancreatic triacylglycerol lipase had significantly higher expression in the CP strains (Additional file 11). Combined with the significantly increased expression of other metabolic genes in CP described above this further suggests a differential role for lipid metabolism in the development of both resting eggs and asexually-produced eggs, as has been suggested previously by gene expression studies specifically examining resting and amictic eggs [24].

### Gene expression in bdelloid rotifers

Using a published gene expression dataset containing 28,922 assembled transcripts from the bdelloid rotifer *A. ricciae*[44], we identified transcripts with significant sequence similarity to the *B. calyciflorus* sequences. Using tblastx with a bit score cutoff of 50, a total of 12,512 (~31%) of the *B. calyciflorus* sequences had at least one significant *A. ricciae* transcript hit (Additional file 14).

We further examined how the proportion of *B. calyciflorus* sequences with significant sequence similarity to *A. ricciae* transcripts changed with the difference in expression observed between CP and OP strains of *B. calyciflorus*. If genes with increased expression in the CP strain represent those that function in a process specific to sexual reproduction, it is likely that no identifiable homolog is present in bdelloids, as selection would no longer maintain the gene for that function. In contrast, genes that show no difference in expression likely have constitutive functions and would be more likely to be conserved in bdelloids. Finally, the presence of genes with higher expression in the OP strain in the *A. ricciae* transcripts would be more likely given that they may function in ameiotic egg production.

**Figure 4:**
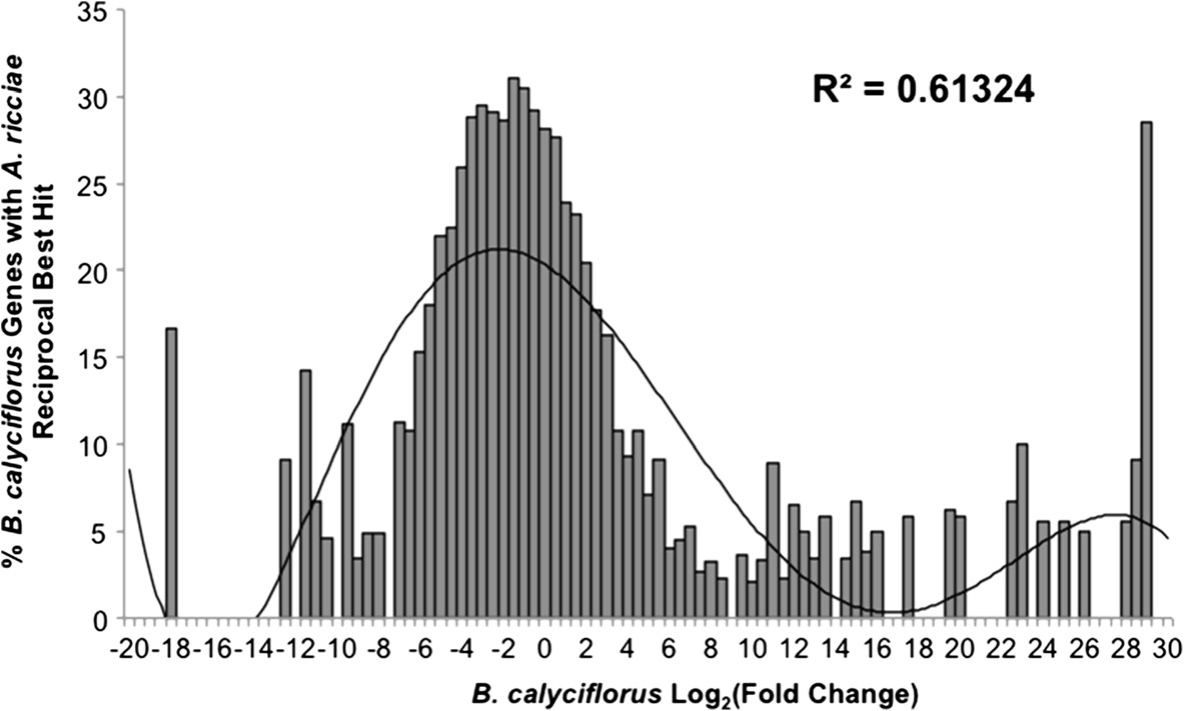
**Distribution of *B. calyciflorus *genes with significant sequence similarity to *A. ricciae *transcripts.** Percentage of *B. calyciflorus* genes with at least one significant *A. ricciae* transcript hit by tblastx (bit score ≥ 50) plotted according to fold change in expression observed between OP and CP strains as calculated by edgeR. R^2^ values for six order polynomial regression analysis given.

The distribution of *A. ricciae* transcripts across the observed CP/OP fold change in *B. calyciflorus* is shown in Figure 4 and Additional file 8. As expected, *B. calyciflorus* genes with little to no change in expression between CP and OP were the most likely to have at least one significant hit in the *A. ricciae* transcripts, with a decrease in the proportion of genes with corresponding bdelloid sequences when expression is higher in the CP strain. However, for genes with the greatest increase in expression in the CP strains, the proportion of sequences with at least one significant *A. ricciae* transcript hit increases. To examine which genes account for this increase in *A. ricciae* transcript hits, a GO term enrichment analysis was performed for the *B. calyciflorus* genes that had both a significant increase in expression in the CP strains and significant sequence similarity to at least one *A. ricciae* transcript (Table 5). The enriched GO terms represent genes with roles in responses to antioxidant stress. Although in monogononts dormancy and desiccation tolerance only occur in resting eggs, bdelloids are capable of entering dormancy at any life stage [15,105]. Notably, the *A. ricciae* transcripts were assembled from a pool of samples obtained from bdelloids under normal conditions as well as various stages of desiccation recovery [44]; thus, expression of genes with functions in dormancy (Additional file 11) may be attributed to the inclusion of transcripts from the recovering samples. However, constitutive expression of genes associated with dormancy cannot be excluded: an extremely efficient antioxidant response has been observed in bdelloids after treatment with ionizing radiation, suggesting such proteins are present to allow for a more rapid response to environmental changes [106].

**Table 5:** Gene ontology enrichment analysis for genes with significant similarity to bdelloid transcript

**GO-ID**	**Term**	**Category**	**FDR**	**P-Value**
GO:0016209	antioxidant activity	F	2.51E-05	1.88E-09
GO:0016491	oxidoreductase activity	F	3.56E-03	5.34E-07
GO:0055114	oxidation-reduction process	P	4.59E-03	1.46E-06
GO:0004601	peroxidase activity	F	4.59E-03	1.72E-06
GO:0016684	oxidoreductase activity, acting on peroxide as acceptor	F	4.59E-03	1.72E-06
GO:0008199	ferric iron binding	F	5.66E-03	2.55E-06
GO:0006950	response to stress	P	6.69E-03	3.51E-06
GO:0008430	selenium binding	F	2.05E-02	1.38E-05
GO:0006979	response to oxidative stress	P	2.05E-02	1.38E-05

Analysis performed for *B. calyciflorus* genes with higher expression in CP strains that have significant sequence similarity to at least one *A. ricciae* transcript. *F* molecular function, *C* cellular component, *P* biological process.

A decrease in the proportion of *B. calyciflorus* genes with significant sequence similarity to *A. ricciae* transcripts was also observed for genes with increased expression in the OP strain (Figure 4, Additional file 6). While this could suggest a larger trend for more highly regulated genes in monogononts having faster rates of evolution or species-specificity, it may also suggest that the genes associated with asexual reproduction have roles in the suppression of sexual processes; these genes would be unnecessary in the bdelloids, as they are not known to undergo the reproductive switch that occurs in monogononts.

Finally, we examined the identified genes in *B. calyciflorus* with known roles in mixis induction, meiosis, and gametogenesis that had significant sequence similarity to *A. ricciae* transcripts. Of the seventy-three nuclear receptor superfamily genes identified in *B. calyciflorus*, twenty-eight had at least one significant *A. ricciae* transcript hit (Additional file 12). Three of the receptors with *A. ricciae* hits are differentially expressed between CP and OP strains of *B. calyciflorus*. However, as mentioned above, the highly conserved DNA binding domain of nuclear receptors makes it difficult to differentiate members within the superfamily, so additional studies will be required to determine the roles of these genes in both monogononts and bdelloids.

For genes with potential roles in meiosis, seven of the histone H2A genes identified in *B. calyciflorus* have significant hits in the *A. ricciae* dataset (Table 3). No significant hits were found for H2A.Z, consistent with a previous study suggesting this variant is absent in bdelloids [57]. The three H2A genes that have significant differential expression in *B. calyciflorus* have at least one *A. ricciae* transcript with significant sequence similarity. Of the 122 monogonont genes with known roles in meiosis in model systems, 73 have *A. ricciae* transcripts, including 31 genes with roles in chromosome structure and interaction, 16 genes involved in recombination, and 26 genes involved in cell cycle progression (Additional file 11). CDC20A, which is differentially expressed in *B. calyciflorus* CP and OP strains, does not have a significant *A. ricciae* transcript hit, although *A. ricciae* transcripts were identified for two other copies, CDC20C and CDC20D. For “meiosis-specific” genes, no *A. ricciae* transcripts were identified with the exception of the MutS homologs Msh4 and Msh5. These genes act as a heterodimer in the resolution of the double-Holliday junction during meiotic recombination [107], and have also previously been identified in bdelloids *via* degenerate PCR (Schurko et al., unpublished).

Of the 39 genes with putative functions in male gametogenesis, twenty-two have significant hits in the *A. ricciae* transcripts (Additional file 13). The presence and expression of these genes in bdelloids combined with the lack of significant differential expression between CP and OP strains of *B. calyciflorus* suggests that roles for these genes are not exclusive to spermatogenesis in rotifers.

## Conclusions

We identified a large set of genes with significantly higher expression in either cyclically parthenogenetic (CP) or obligately parthenogenetic (OP) populations of the monogonont rotifer, *Brachionus calyciflorus*. Included in this set of genes are those with potential roles in processes specific to sexual reproduction in monogononts such as mixis induction, meiosis and gametogenesis, and dormancy. We further identified several genes with increased expression in OP populations that suggest developmental and metabolic differences during asexual egg production. The samples used in this study included entire populations of monogonont rotifers, and future studies focusing on specific life cycle stages will clarify and enhance the data presented here.

Notably, none of the identified “meiosis-specific” genes were differentially expressed between OP and CP populations (Additional file 11). Although the expression levels were low for some of these genes—potentially due to restricted temporal and spatial expression to meiosis, which is likely only occurring in a subset of embryos in the population [108]—this is consistent with observations made in previous studies suggesting that these genes are not always robust markers for sex, as their transcription is not specific to meiosis in all organisms. For example, in the cyclical parthenogen *Daphnia pulex*, these genes are expressed in the ovaries of both sexual and asexual females [109]. Furthermore, oogenesis in asexual *D. pulex* females is an abortive meiotic division, suggesting that some of these genes may be required for egg production regardless of reproductive status [110].

Examples such as *D. pulex* highlight the importance of an unbiased examination of global gene expression patterns in establishing markers for sexual reproduction in rotifers. Our transcriptome analysis of *B. calyciflorus* reveals differential expression of genes that have roles in sexual processes in other systems as well as many genes that have no identifiable homologs in NCBI that may represent fast evolving or rotifer-specific genes. With this more informed frame of reference, we have provided an initial analysis of bdelloid transcriptome data that provides us with unique insights relating to the molecular nature of long-term asexual egg production and/or any potential for meiosis or the inclusion of meiotic processes during oogenesis in bdelloids.

## Competing interests

The authors declare that they have no competing interests.

## Authors’ contributions

SJH designed and carried out molecular studies, performed data analysis and drafted the manuscript; CPS contributed to experimental design; DBMW conceived of the study and contributed to experimental design; JML conceived of the study and contributed to experimental design and coordination; all authors edited the manuscript. All authors read and approved the final manuscript.

## Supplementary Material

Additional file 1 Assembled transcripts for sample OP1 less than 200 base pairs in length. Click here for file

Additional file 2 Assembled transcripts for sample OP2 less than 200 base pairs in length. Click here for file

Additional file 3 Assembled transcripts for sample CP1 less than 200 base pairs in length. Click here for file

Additional file 4 Assembled transcripts for sample CP2 less than 200 base pairs in length. Click here for file

Additional file 5 Primers used in qRT-PCR. Click here for file

Additional file 6 **OP and CP replicate comparison.** Log_2_FPKM values for A) OP and B) CP replicates plotted. Pearson correlation values (r) given. C) Multi-dimensional scaling analysis performed in edgeR. Click here for file

Additional file 7 **Library validation.** Fold change values determined by quantitative RT-PCR plotted against values calculated in **A**) Cuffdiff or **B**) edgeR. Pearson correlation values (r) given. Click here for file

Additional file 8 **Analysis of gene expression changes. A**) Log_2_ (Fold Change) distribution (CP/OP) is shown as determined by Cuffdiff. **B**) Distribution of *B. calyciflorus* genes with significant sequence similarity to *A. ricciae* transcripts. Percentage of *B. calyciflorus* genes with at least one significant *A. ricciae* transcript hit by tblastx (bit score ≥ 50) plotted according to fold change in expression observed between OP and CP strains as calculated by Cuffdiff. R^2^ values for six order polynomial regression analysis given. Click here for file

Additional file 9 **GO term assignments, BLAST identity, and expression analysis for genes with significant differential expression in Cuffdiff or edgeR between OP and CP *B. calyciflorus*.** Click here for file

Additional file 10 **FPKM box and whisker plots.** Distribution of log (FPKM) values in OP and CP libraries shown for **A**) all expressed genes, **B**) housekeeping genes, **C**) dormancy genes, **D**) full inventory of meiosis genes, **E**) meiosis genes involved in recombination, **F**) meiosis genes involved in chromosome structure and interaction, **G**) meiosis genes involved in cell cycle regulation, and **H**) male gametogenesis genes. Distribution of genes compared by Kolmogorov-Smirnov. N.s. = not significant. Click here for file

Additional file 11 **Housekeeping, meiosis inventory, histone, and dormancy genes identified in OP and CP libraries.** No Test = expression values did not meet threshold required for significance testing. Gene names highlighted in bold have “meiosis-specific” functions. Click here for file

Additional file 12 **Nuclear receptor superfamily members identified in OP and CP libraries.** No Test = expression values did not meet threshold required for significance testing. Click here for file

Additional file 13 **Male gametogenesis genes identified in OP and CP libraries.** No Test = expression values did not meet threshold required for significance testing. Click here for file

Additional file 14 **BLAST results for *A. ricciae *transcripts against *B. calyciflorus *genes.** Click here for file
