# Supplementary figures and images for: Comparative transcriptome analysis of obligately asexual and cyclically sexual rotifers reveals genes with putative functions in sexual reproduction, dormancy, and asexual egg production
Source: BMC Genomics. 2013 Jun 19;14:412. doi: 10.1186/1471-2164-14-412 (PMC3701536; doi:10.1186/1471-2164-14-412)

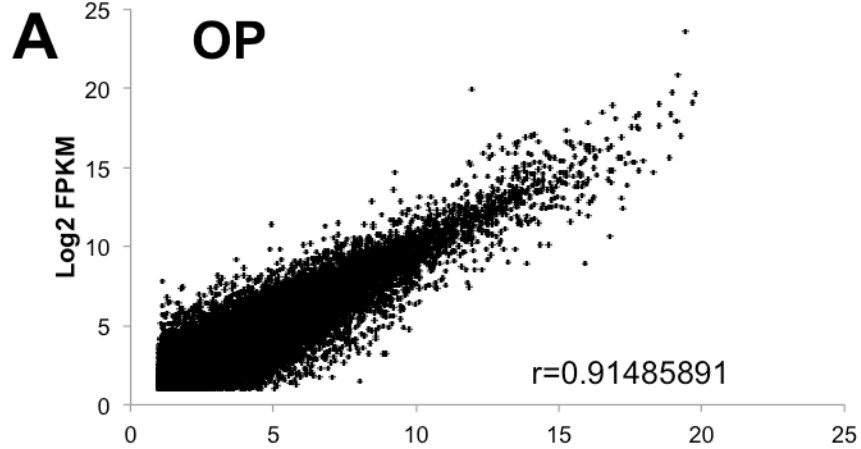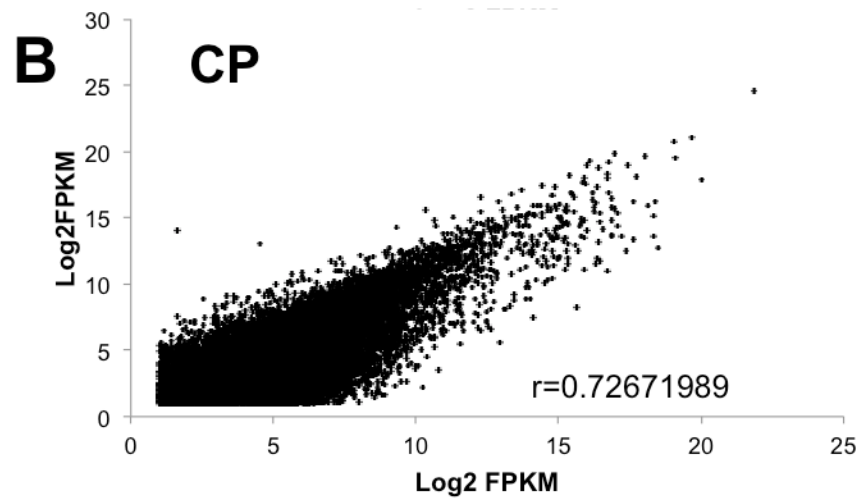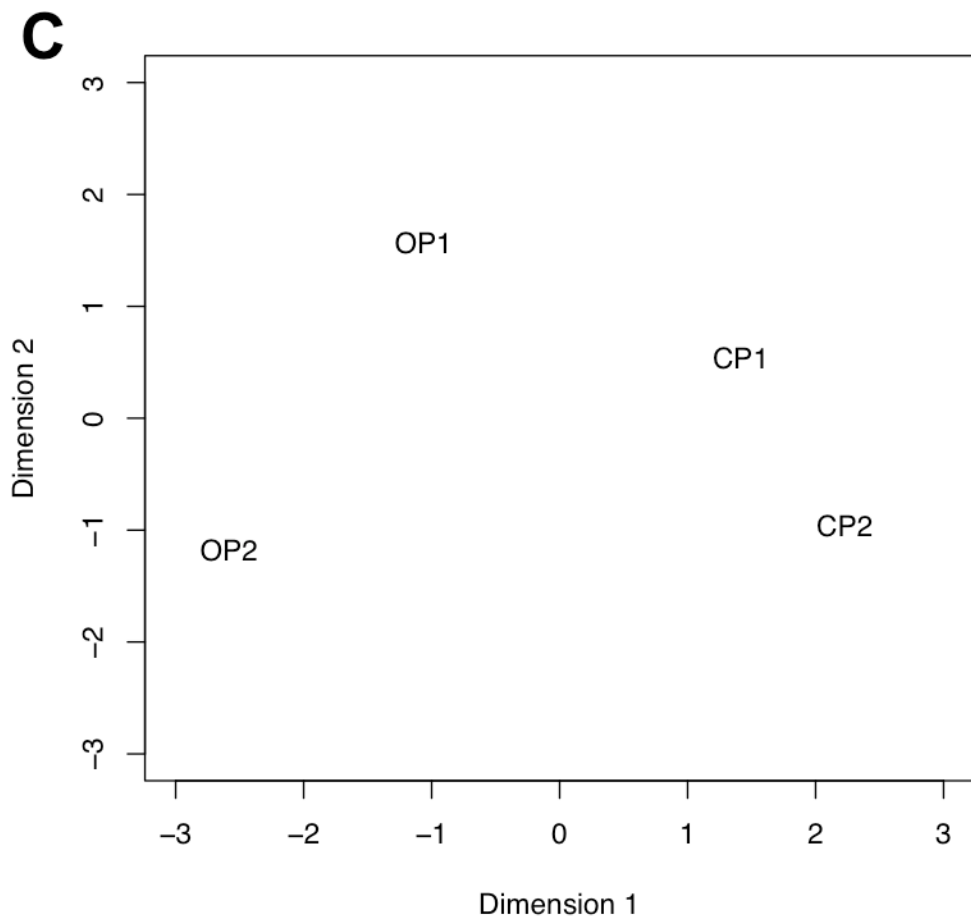

Supplement: Additional file 6 — OP and CP replicate comparison. Log2FPKM values for A) OP and B) CP replicates plotted. Pearson correlation values (r) given. C) Multi-dimensional scaling analysis performed in edgeR. [file 1471-2164-14-412-S6.pdf]

**A**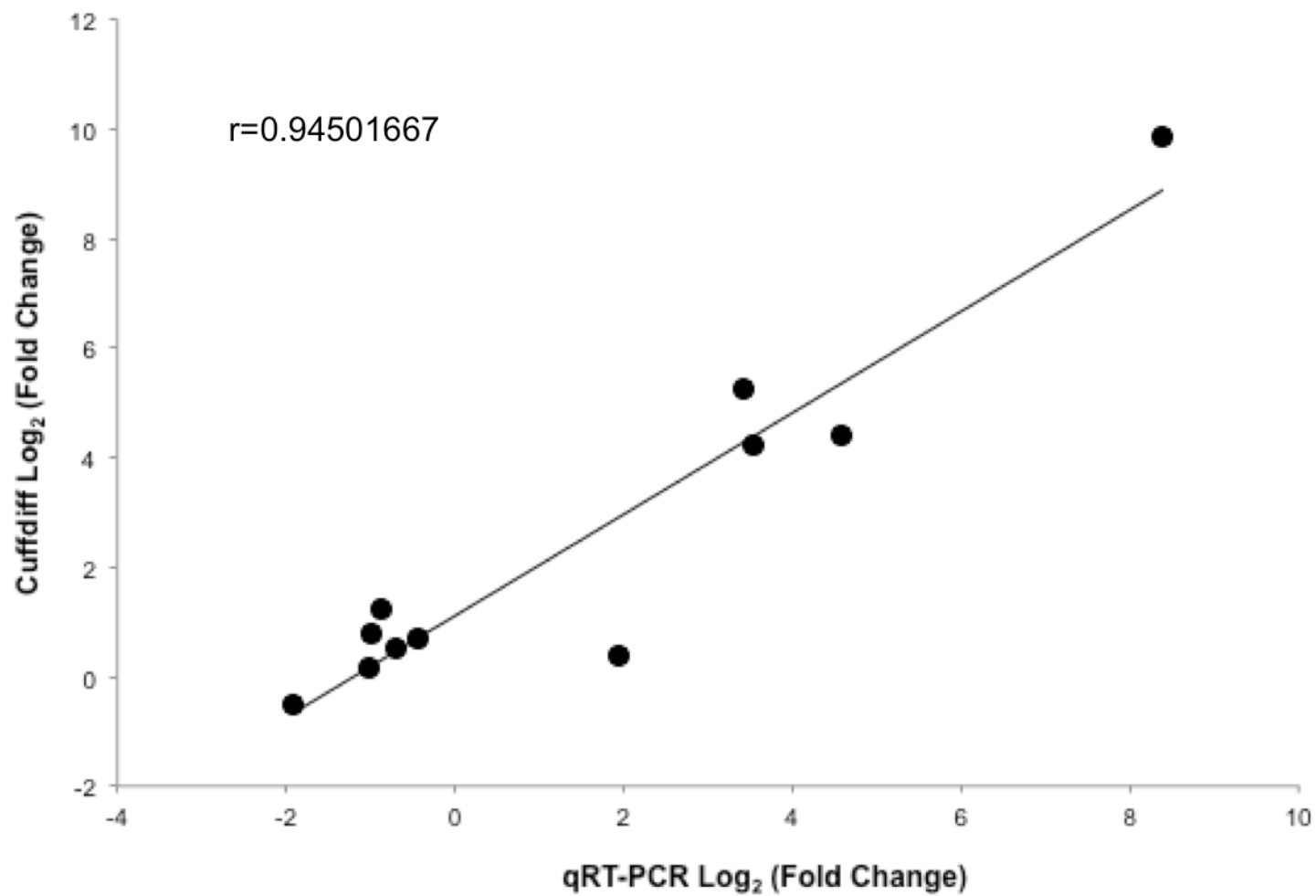**B**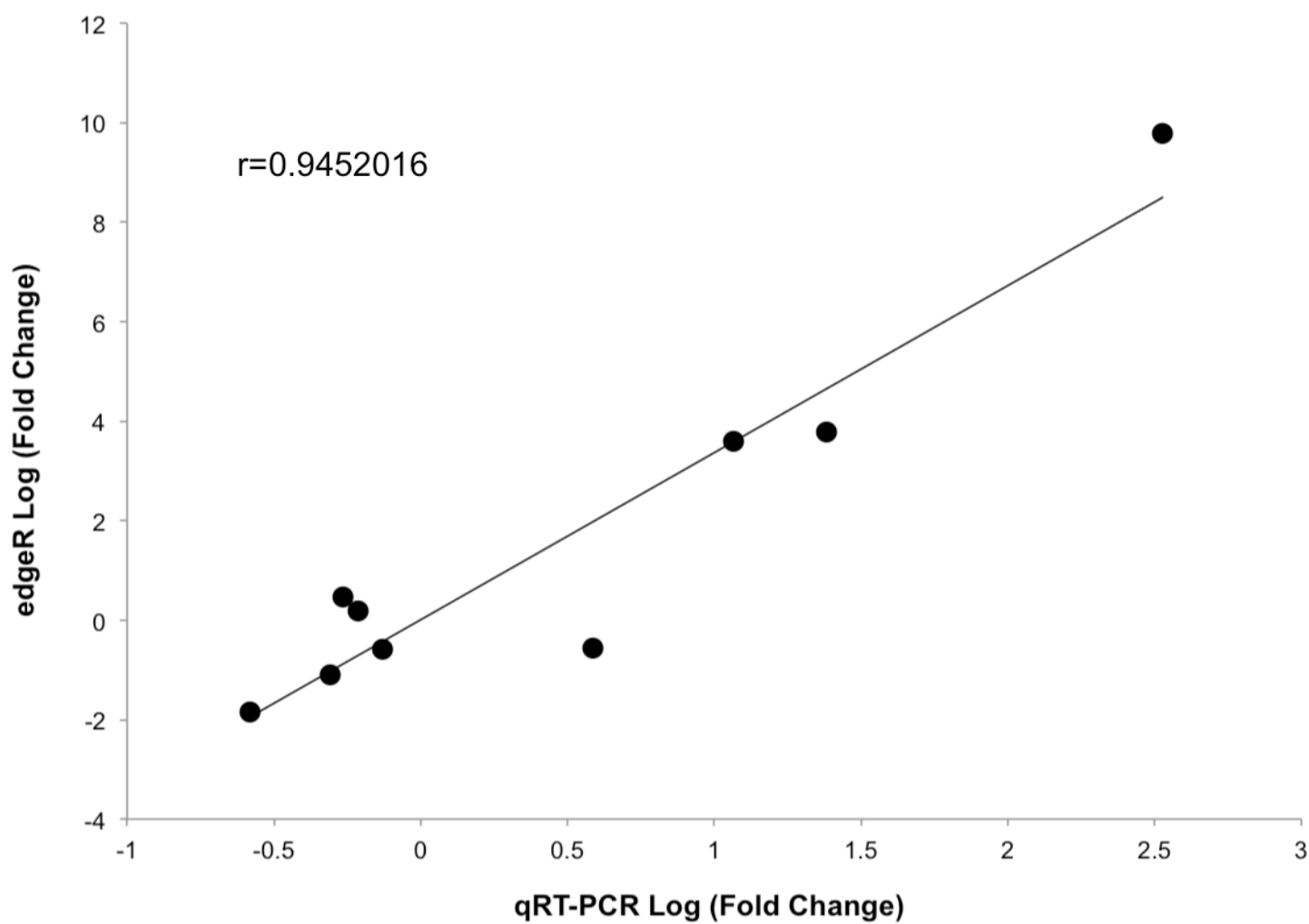

Supplement: Additional file 7 — Library validation. Fold change values determined by quantitative RT-PCR plotted against values calculated in A) Cuffdiff or B) edgeR. Pearson correlation values (r) given. [file 1471-2164-14-412-S7.pdf]

**A**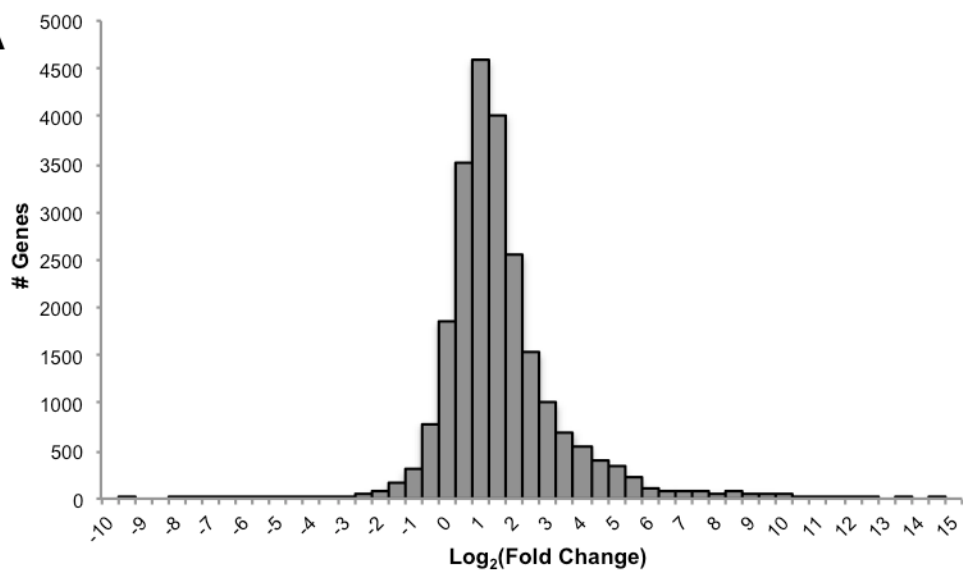**B**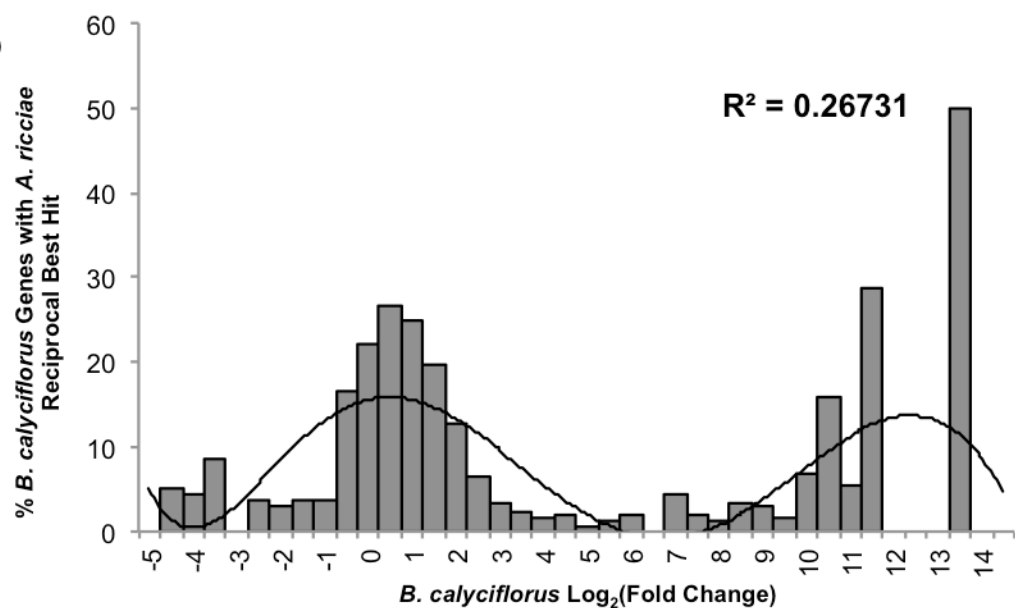

Supplement: Additional file 8 — Analysis of gene expression changes. A) Log2 (Fold Change) distribution (CP/OP) is shown as determined by Cuffdiff. B) Distribution of B. calyciflorus genes with significant sequence similarity to A. ricciae transcripts. Percentage of B. calyciflorus genes with at least one significant A. ricciae transcript hit by tblastx (bit score ≥ 50) plotted according to fold change in expression observed between OP and CP strains as calculated by Cuffdiff. R2 values for six order polynomial regression analysis given. [file 1471-2164-14-412-S8.pdf]

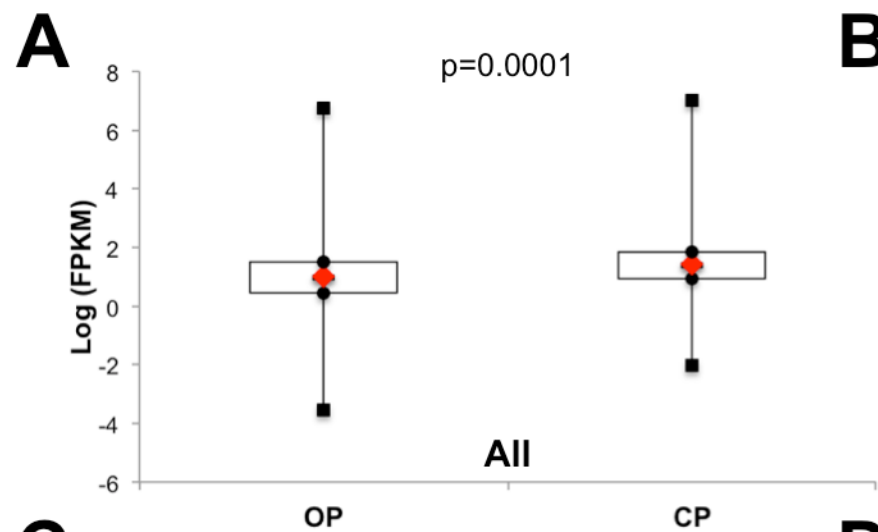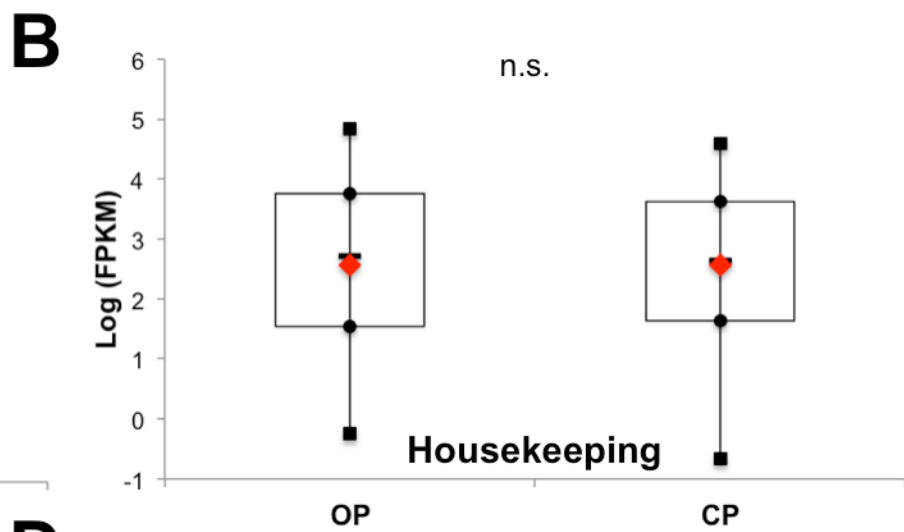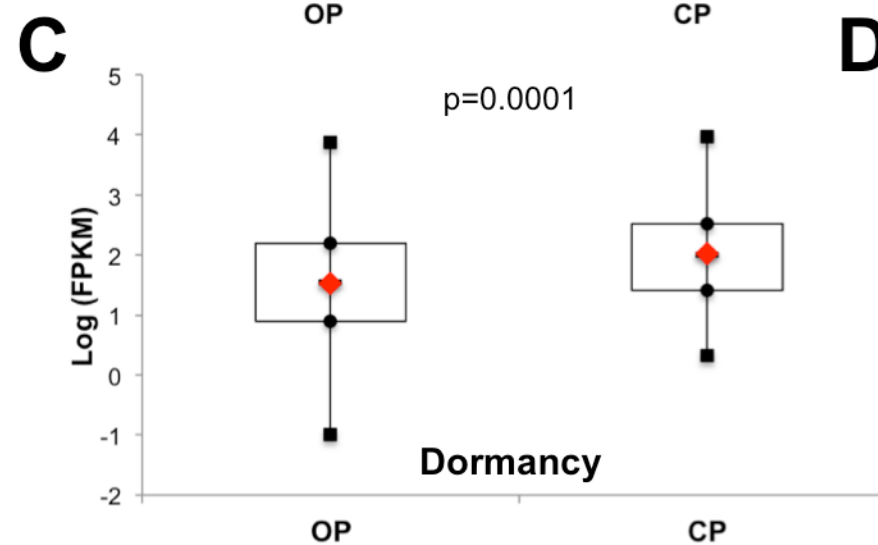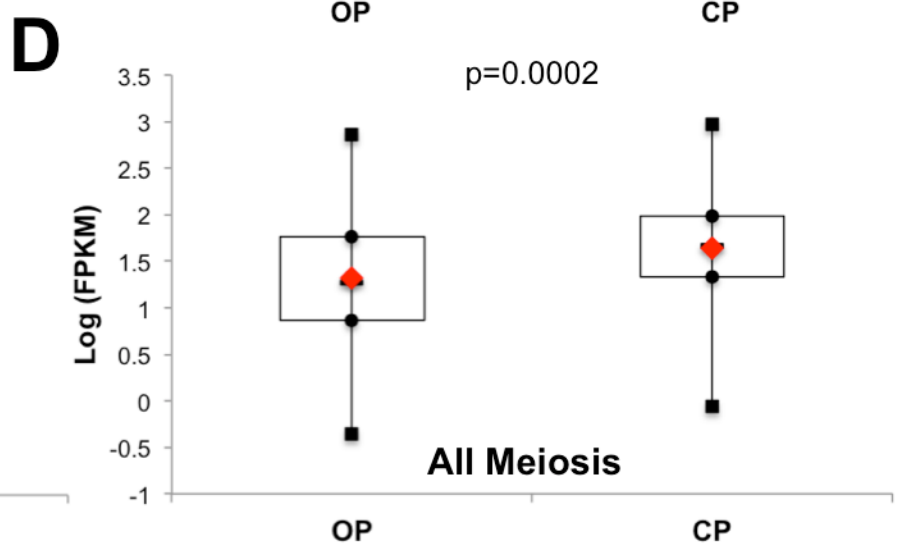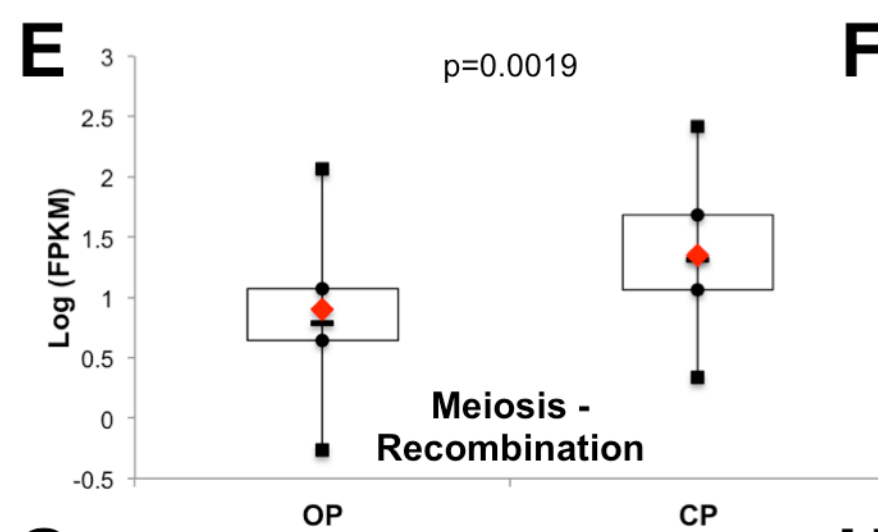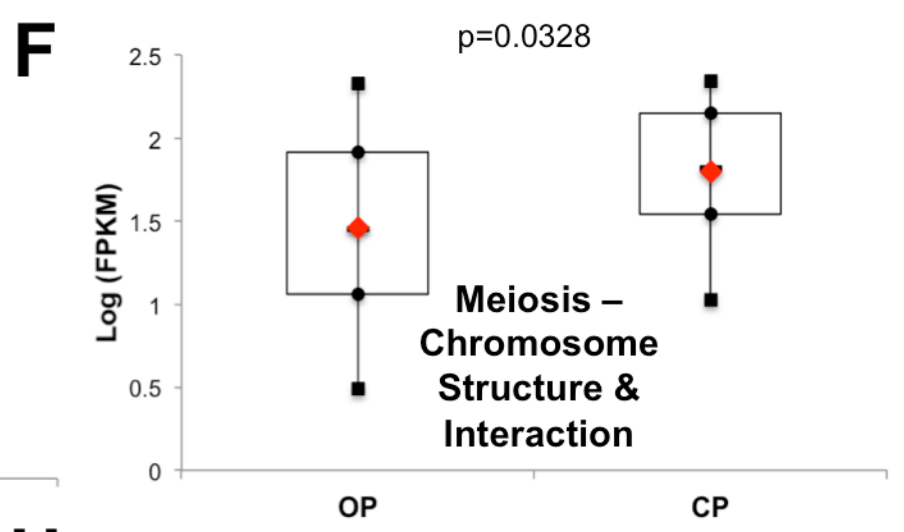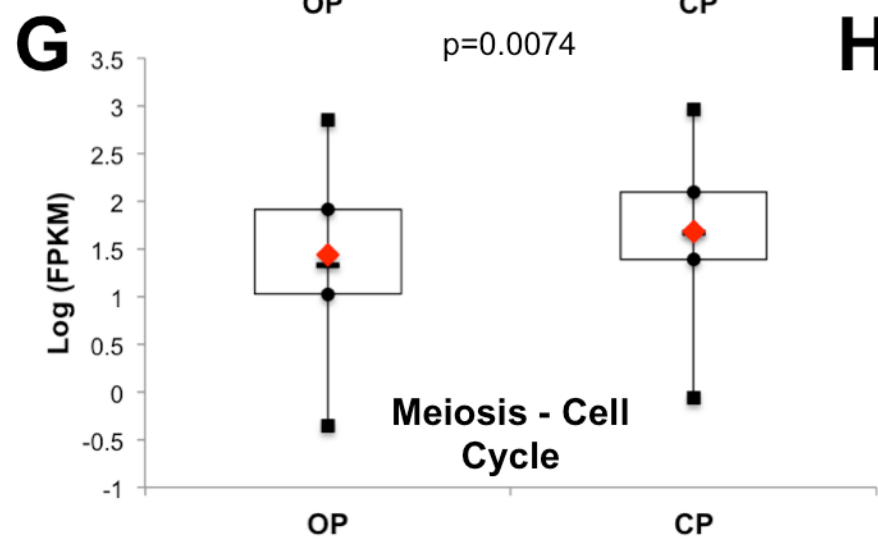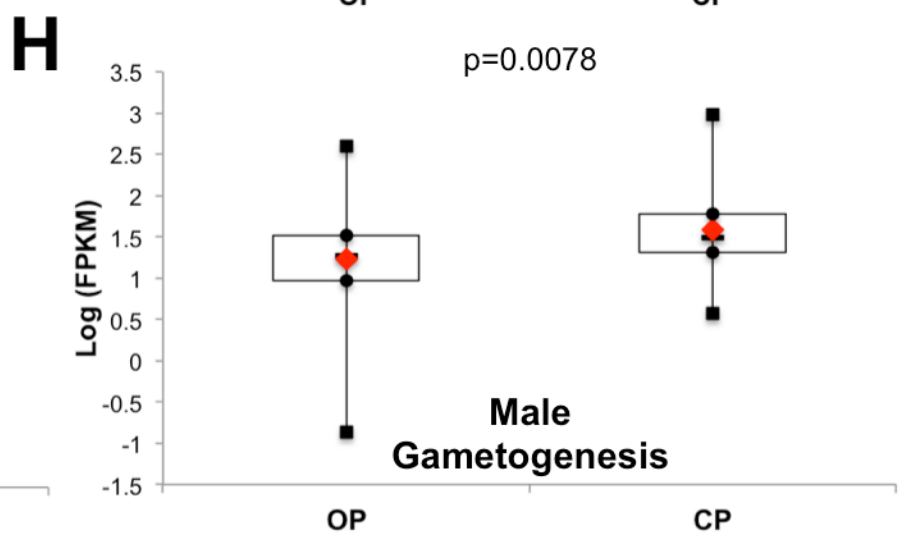

Supplement: Additional file 10 — FPKM box and whisker plots. Distribution of log (FPKM) values in OP and CP libraries shown for A) all expressed genes, B) housekeeping genes, C) dormancy genes, D) full inventory of meiosis genes, E) meiosis genes involved in recombination, F) meiosis genes involved in chromosome structure and interaction, G) meiosis genes involved in cell cycle regulation, and H) male gametogenesis genes. Distribution of genes compared by Kolmogorov-Smirnov. N.s. = not significant. [file 1471-2164-14-412-S10.pdf]
